# Gastrointestinal involvement in Klippel-Trénaunay syndrome: pathophysiology, evaluation, and management

**DOI:** 10.1186/s13023-023-02857-5

**Published:** 2023-09-12

**Authors:** Huaijie Wang, Weilong Lin, Chong Xie, Weijia Yang, Jinbang Zhou, Zhengtuan Guo

**Affiliations:** Department of Pediatric Surgery & Vascular Anomalies, Xi’an International Medical Center Hospital, 710100 Xi’an, China

**Keywords:** Klippel-Trénaunay syndrome, Bleeding, Venous malformation, Portal hypertension, Sclerotherapy, Rex Shunt, Sigmoidectomy

## Abstract

Klippel-Trénaunay syndrome is typically a complex combined capillary-lymphatic-venous malformation in lower limb. Gastrointestinal involvement is not infrequent in Klippel-Trénaunay syndrome. Rectal bleeding is the most common complication. In recent years, this condition has been increasingly reported. However, most authors simply described extreme manifestations or various combinations of clinical observations. The underlying pathophysiology of gastrointestinal involvement in Klippel-Trénaunay syndrome has been underrecognized. Pathophysiologically, some seemingly adequate managements are pitfalls in treatment. Anorectosigmoid vascular malformations in KTS have distinct and more complicated pathophysiologies than anorectal vascular malformation. Once understanding the pathophysiology, some patients can be successfully managed with a staged plan in our practice. Therefore, recognizing the pathophysiologies of gastrointestinal involvement is needed to evaluate, prevent pitfalls, and determine adequate managements for practitioners. Because of the complexity and rarity of this condition, prospective controlled study or a large cohort of patients is impossible. Based on literature review and our practice, we discuss pathophysiologies, evaluation, pitfalls, and treatment strategies for gastrointestinal involvement in Klippel-Trénaunay syndrome.

## Introduction

Klippel-Trénaunay syndrome (KTS) is a congenital combined capillary-lymphatic-venous malformation in an overgrown limb [1]. KTS typically involves lower extremity unilaterally, occasionally occurs in the upper extremity or both legs [1]. It can feature as a complex of vascular malformations involving a lower limb, pelvis and abdomen [1, 2]. Gastrointestinal involvement is common in KTS patients, which can be a source of significant morbidity and even mortality [1–3]. Its incidence is underestimated, may be as high as over 30% in patients with KTS [2, 4]. Gastrointestinal involvement is typically presented with edematous and thickened colorectum by the venous malformation (VM) network around and intra-wall [3, 5, 6]. These VMs can cause recurrent rectal bleeding, ranging from occult to massive, and life-threatening hemorrhages [1, 2]. Conservative management has been successfully used in many patients to manage iron deficiency anemia from bleeding [7]. Invasive treatments included surgical resection of involved bowel [4, 7–11], and interventional approaches [2], and increasingly reported sclerotherapy [12–14].

Many gastrointestinal VM patients with KTS have been reported, however, most authors only simply illustrated extreme manifestations or depicted various interesting combinations of signs, symptoms, laboratory parameters, and images of disease [15–31]. Since the complexity and underrecognized pathophysiology of KTS with pelvis and gastrointestinal involvement, various treatments have not been integrated into a whole management strategy. There are no current recommendations for managing of gastrointestinal VM in KTS. In some instance, some seemingly adequate treatments may be contraindicated [3, 6]. For example, a popular treatment, sclerotherapy can be preferred for anorectal VM managing [2, 3, 6], but primary sclerotherapy is not suitable for anorectosigmoid VM in KTS, since norectosigmoid VM has distinct pathophysiologies from anorectal VM [3].

KTS can also affect spleen and portal vein system [1, 3, 20, 22, 32–36]. Generally, several treatments can be used to treat portal hypertension, including portosystemic shunt or mesenterico-Rex shunt [37, 38], however, primary shunt may be contraindicated if the inferior mesenteric vein (IMV) involved in KTS [3]. Practitioners need to be aware of the underlying pathophysiology of gastrointestinal VM in KTS patients, in order to improve managements. We discuss the pathophysiologies, pitfalls, and propose evaluation and treatment strategy that can help in managing KTS with gastrointestinal VM.

### Pathophysiology and management of anorectal VM

**Fig. 1:**
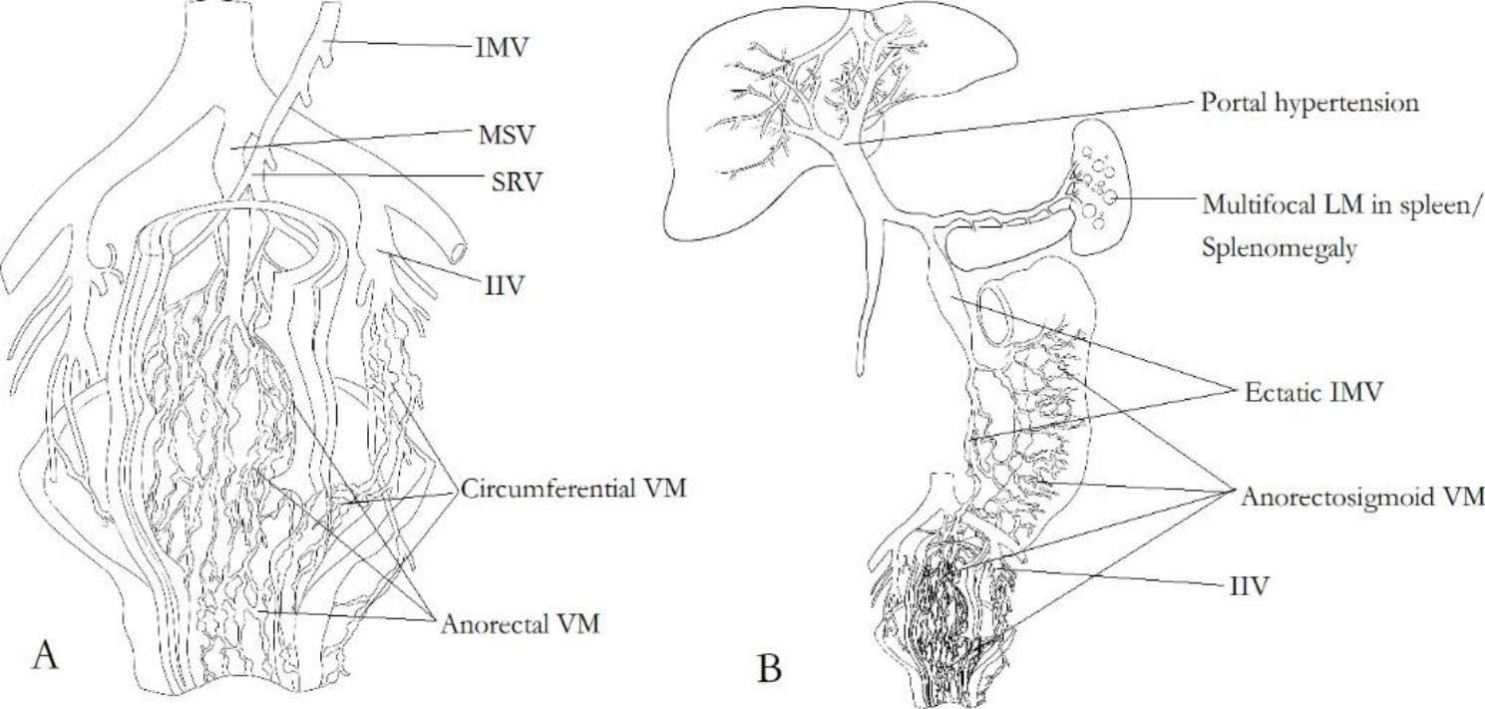
The pathophysiologies of anorectal and anorectosigmoid VM in KTS. Panel **A**: Anorectal and circumferential VM mainly drain into internal iliac vein (IIV), rarely into median sacral vein (MSV). It is noteworthy that the superior rectal vein (SRV) and inferior mesenteric vein (IMV) are not affected. Panel **B**: Anorectosigmoid VM drains into the IIV, the SRV and IMV, which means that the IMV and portal vein system is involved. Therefore, ectatic IMV and portal hypertension can be identified in patients. The latter is partly caused by the thrombi within VM migrating up into and obstructing the branches of portal vein via the IMV. Multifocal lymphatic malformation (LM) in spleen also can be seen in KTS patients

In current literature and our practice, gastrointestinal involvement in KTS can be mainly categorized into anorectal and anorectosigmoid VM (Fig. 1). Defined, more extensive colon involvement has rarely been reported and not been encountered by us in KTS patients.

**Fig. 2:**
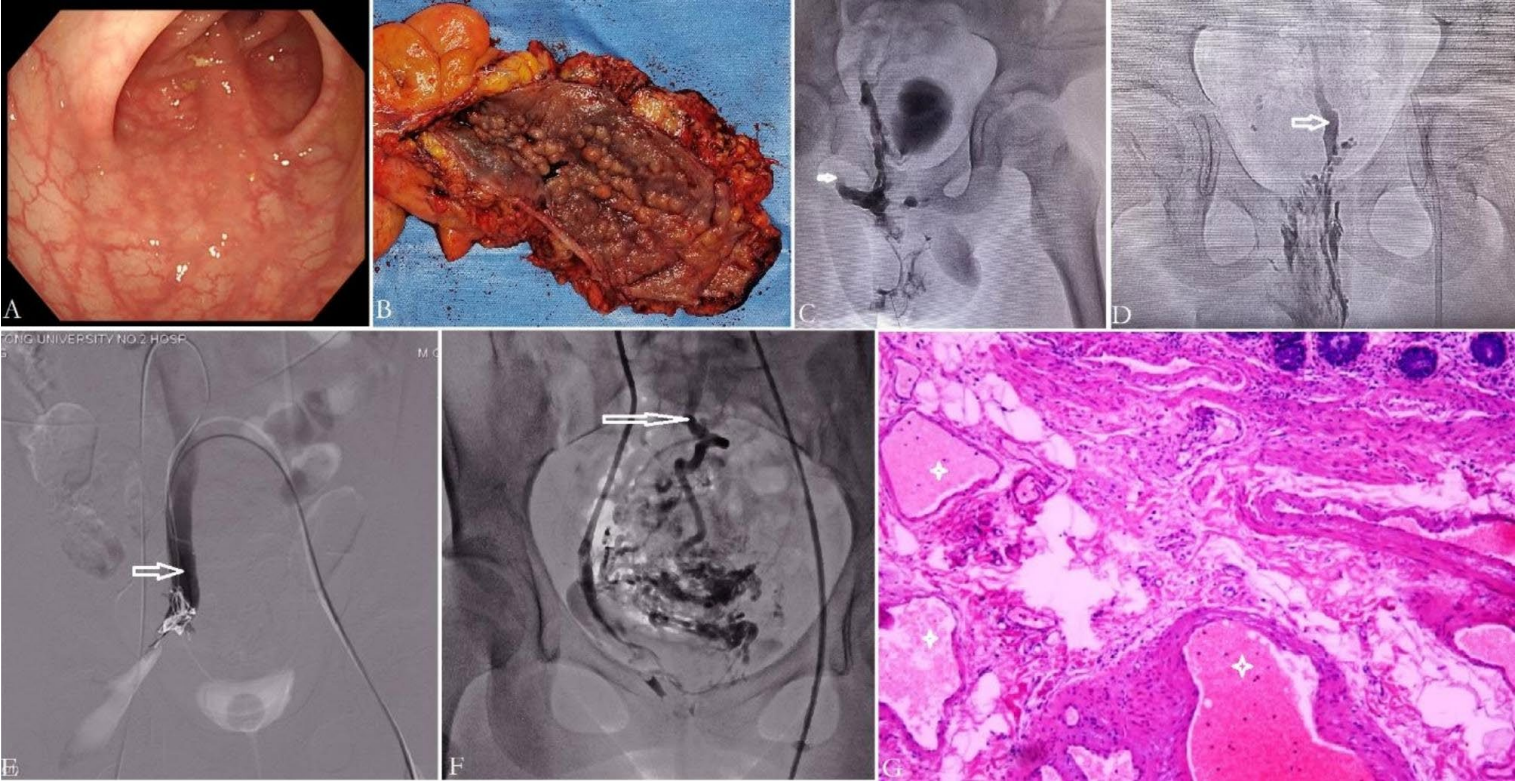
Clinical findings in KTS with anorectal and anorectosigmoid VM. VM develops with age, featuring as submucosal reticular phlebectasia in early stage (Panel **A**), and edematous, stiff and thickened gut by full thickness wall involvement in late stage (Panel **B**). Active bleeding sites usually can not be identified endoscopically. When sclerotherapy via imaging-guided direct puncture, we can see anorectal VM drains into internal iliac vein (IIV) (Panel **C**) (arrow), and/or into median sacral vein (Panel **D**) (arrow). Panel **E** shows trans-IIV (arrow) sclerotherapy for ablating rectal bleeding. Panel **F**: In a KTS patient with anorectosigmoid VM, trans-IIV phlebography demonstrated that part of VM drained into the superior rectal vein (arrow), which is indicative of portal vein system involvement. Panel **G**: Histopathologically, VM are dilated, thin-walled, sponge-like abnormal channels. Extensive venous thrombosis (asterisk) can be identified within the wall of affected colon

In our study, internal iliac vein (IIV) malformation and reflux has been identified in KTS patients with pelvis involvement [2]. In these patients, the IIV-gluteal-marginal vein system is a valveless malformed reflux channel [2]. IIV reflux leads to local venous hypertension in pelvic organs, including rectum and genitourinary tracts [2]. Because of chronic vein hypertension, anorectal VM develops slowly with age, manifesting as submucosal reticular phlebectasia of anorectum in children (Fig. 2), and edematous, stiff and thickened rectum by full thickness wall involvement in adults (Fig. 2).

**Fig. 3:**
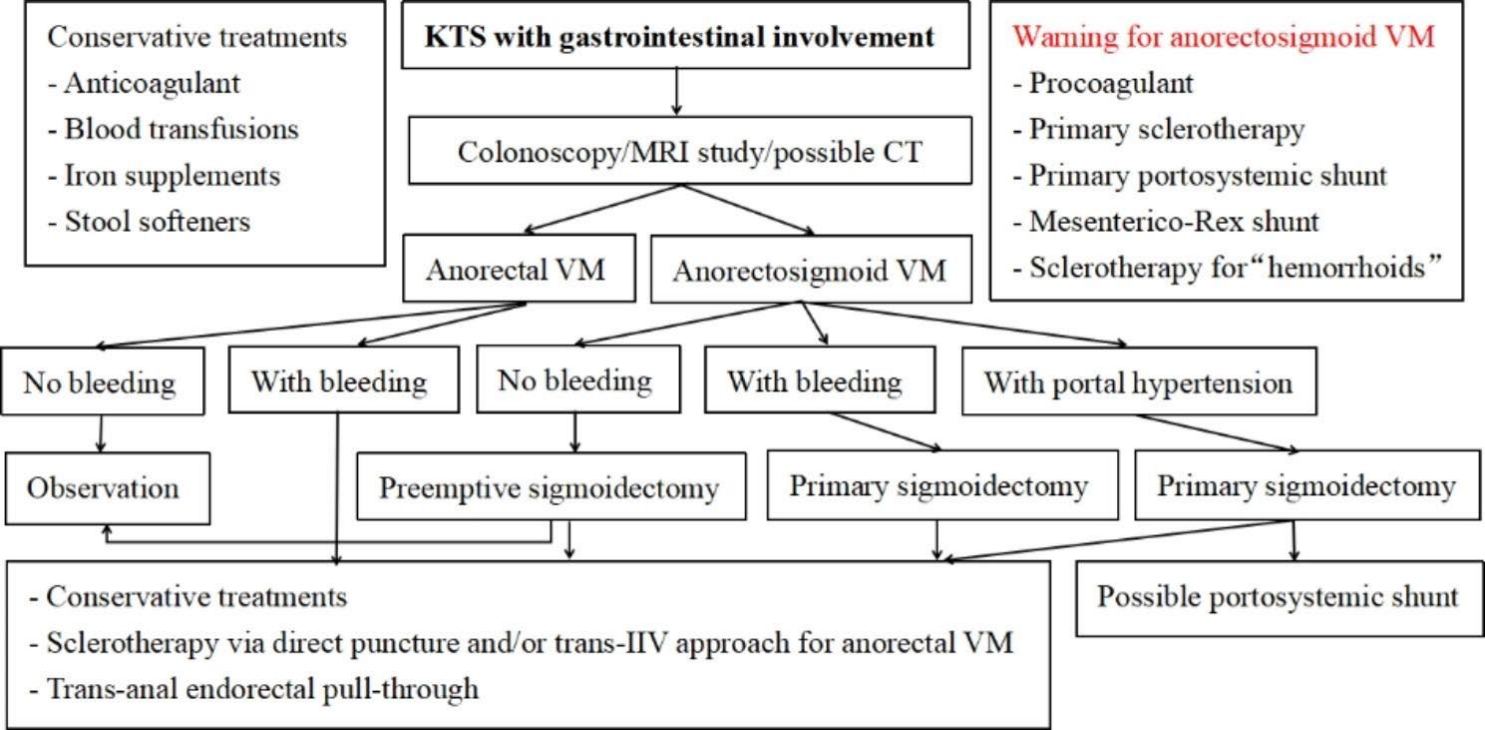
Treatment algorithm for gastrointestinal involvement in KTS.

The rupture of submucosal phlebectasia leads to rectal bleeding from VM. Its management is initially conservative, including anticoagulant, blood transfusions, iron supplements, sirolimus, and stool softeners [7] (Fig. 3). However, anorectal VM with clinically significant hemorrhages usually requires invasive managements [2].

Surgical resection with endorectal pull-through is often safe and feasible in children [5, 8–10]. Anorectal VM develops with age, however, surgical resection is usually difficult and risky in adults, carrying the risk of problematic bleeding [20]. Sclerotherapy is becoming a popular management recently. Since anorectal VM mainly drains into the IIV [2, 3, 6, 39], and rarely into the median sacral vein [39] (Figs. 1 and 2), adequate sclerotherapy carries little complication [3, 12, 14]. It is a safe choice and recommended by clinical practice guideline [1]. We prefer ethanol-based sclerotherapy via direct puncture and/or trans-IIV approaches [2, 3, 6] (Figs. 2 and 3).

### Pathophysiology of anorectosigmoid VM and portal hypertension

Anorectosigmoid VM has distinct pathophysiologies from anorectal VM [3]. Firstly, it can cause portal hypertension in KTS patients [3, 35, 36] (Fig. 1). Anatomically, the upper section of rectum and sigmoid colon drain into the IMV. So, the VM can drain into two systems, IIV and IMV (Figs. 1 and 2). The latter belongs to the portal vein system. Ectatic, incompetent IMV has been identified in colorectal VM [3, 34, 35]. Dual reflux from IIV and IMV, or stagnant flow in rectosigmoid VM and mesenteric veins can predispose to thrombosis and propagation of thrombus (Fig. 2). Primary VM along, around and within the rectosigmoid wall serves as a spongy blood pool and the source of thrombus [3, 6] (Fig. 2). Once thrombi migrate up into and obstruct the branches of portal vein via the IMV, portal vein thrombosis ensues [3, 35]. Initial portal vein thrombosis can occur in utero in patients with IMV involvement [34]. Secondly, local hypertension within VM from IIV reflux furthers thrombosis and embolus migrating towards IMV. Thirdly, siphon venous flow from incompetent mesenteric veins can lead to reduced venous flow, aggravated stagnant and resultant thrombosis extending in the portal vein. Finally, portal hypertension also worsen anorectosigmoid VM and bleeding [3]. These portosystemic (IIV-IMV) interactive mechanisms are responsible for the progress of disease. Naturally, patients may develop evident cavernous transformation, persistent, aggravated rectal bleeding, gastroesophageal varices, and ascites.

Portal hypertension has been reported in KTS patients [20, 23, 34–36], but the underlying pathophysiology we discussed above is underrecognized in literature. The IMV, a usually overlooked vein system in patients with portal hypertension should be re-looked in patients with anorectosigmoid VM.

### Pitfalls and recommendations in the management of anorectosigmoid VM and portal hypertension

#### Anticoagulants rather than procoagulants should be administered to manage anorectal bleeding in KTS with colorectal involvement

A chronic coagulation disorder, localized intravascular coagulopathy (LIC) is common in VM, featuring by elevated D-dimer level, low fibrinogen level and variable platelet count [40, 41]. LIC can be also seen in KTS patients with colorectal VM [42]. Blood stagnation within the VM leads to constant activation of coagulation, then causes the thrombi producing and the fibrinogen converting into fibrin. The subsequent fibrinolysis is interpreted by elevated levels of D-dimer and fibrinogen degradation products. Such activation process results in localized coagulopathy within VM. LIC can cause localized bleeding and/or thrombosis, even coagulation factors and platelet levels are normal. LIC is chronic and usually well tolerated in daily life, but trauma, sclerotherapy, and surgery can induce markedly localized consumption of platelets, fibrinogen, and coagulation factors. This process can trigger systemic activation of coagulation, leading to the progression of LIC to disseminated intravascular coagulation (DIC). Increased prothrombin time and persist bleeding from surgical site can be the index.

So, procoagulants play deleterious roles in the bleeding under this condition. Anticoagulant, such as rivaroxaban and low-molecular-weight heparin, can be used to improve the LIC associated bleeding and to prevent procedure induced conversion of LIC towards DIC [40, 41]. In our experience, preprocedural anticoagulants could improve the bleeding and blood parameters change of LIC. Additionally, anticoagulant may be potentially beneficial for portal hypertension [37, 38, 43].

#### Primary sclerotherapy should be avoided in KTS with anorectosigmoid involvement

As the aforementioned pathophysiologies, sclerosants and induced clots can flow into the portal vein via IMV when sclerotherapy is primarily used to treat anorectosigmoid VM in KTS [3, 6] (Fig. 1). This process may induce and/or exacerbate potential portal vein thrombosis and/or portal hypertension [3]. Acute portal vein thrombosis can occur after endovenous treatment [44].

#### Primary sigmoidectomy is recommended in KTS with anorectosigmoid involvement

Primary sigmoidectomy is recommended for managing anorectosigmoid involvement in KTS regardless of bleeding. This surgery can ablate the primary VM lesion, source of original thrombus, and cut off the migrating channel (IMV and superior rectal vein) of thrombus into portal vein [3]. Then, portal hypertension can be stabilized or improved. This surgery also ablates potential bleeding sites and may cure blood loss from colon [3]. After this surgery, the subsequent treatments for residual VM in anorectum are just like the management for anorectal VM aforementioned [3] (Fig. 3). Trans-anal endorectal pull-through operation can be a choice for managing residual VM in anorectum (Fig. 3). Also, sclerotherapy can be safely performed, carrying a very little risk of portal vein issues since the lesion drains into systemic vein system [3, 6] (Fig. 1). Sclerotherapy can be performed via direct puncture and/or trans-IIV approaches [2, 3] (Fig. 3). Consequently, staged operations and intervention can be considered for managing anorectosigmoid involvement in KTS (Fig. 3).

Considering the potential causative role of anorectosigmoid VM in portal hypertension, preemptive sigmoidectomy is recommended to prevent the development of portal hypertension complications and downstream sequelae (Fig. 3).

#### Primary portosystemic shunt should be avoided in KTS with anorectosigmoid involvement and portal hypertension

Pulmonary embolism can occur spontaneously in 4% KTS patients, which cause chronic thromboembolic pulmonary hypertension if recurrent [7, 45]. In patients with portal hypertension related complications, some practitioners may consider primary portosystemic shunt. However, this shunt may be not suitable for portal hypertension in KTS patients [3]. Following primary portosystemic shunt, thrombi in the rectosigmoid VM, IMV system and portal system can migrate into the systemic vein via the shunt channel, which may cause/worsen potential chronic thromboembolic pulmonary hypertension if recurrent [3]. It has been reported KTS patient with congenital extrahepatic portosystemic shunt developed severe pulmonary hypertension and subsequently underwent surgical shunt ligation [46]. In this patient, both IIV and IMV systems were involved. After portosystemic shunt ligation, pulmonary hypertension was greatly improved [46]. This case report addresses the risk of pulmonary hypertension caused by primary portosystemic shunt if IMV system involved in KTS.

As the above pathophysiology of anorectosigmoid VM and portal hypertension (Fig. 1), sigmoidectomy should be considered primarily (Fig. 3), then portosystemic shunt can be performed since the primary VM, source of original thrombus has been removed [3]. Staged portosystemic shunt carries little risk of pulmonary issues. So staged procedures can be planned to improve portal hypertension in selected KTS patients (Fig. 3).

#### Mesenterico-Rex shunt is not suitable for cavernous transformation of the portal vein in KTS patients

Mesenterico-Rex shunt is usually used to manage extra-hepatic portal vein obstruction in children [37, 38, 43]. Extra-hepatic portal venous obstruction is the commonest cause of prehepatic portal hypertension in children [47]. Most have no obvious etiologic cause in their history, may be idiopathic or congenital. In this form of portal hypertension, the obstruction seems to occur in the trunk, leaving the branches patent in liver [47]. The intrahepatic portal system is usually hypoplastic with small-diameter, and may not be visible on conventional imaging [47]. Mesenterico-Rex shunt is to construct a bypass between the extra-hepatic portal vein and the left portal vein in Rex recessus to reconstruct the hepatopetal portal blood [38, 43]. So, this shunt can decompress portal hypertension and possibly cure most of these children.

**Fig. 4:**
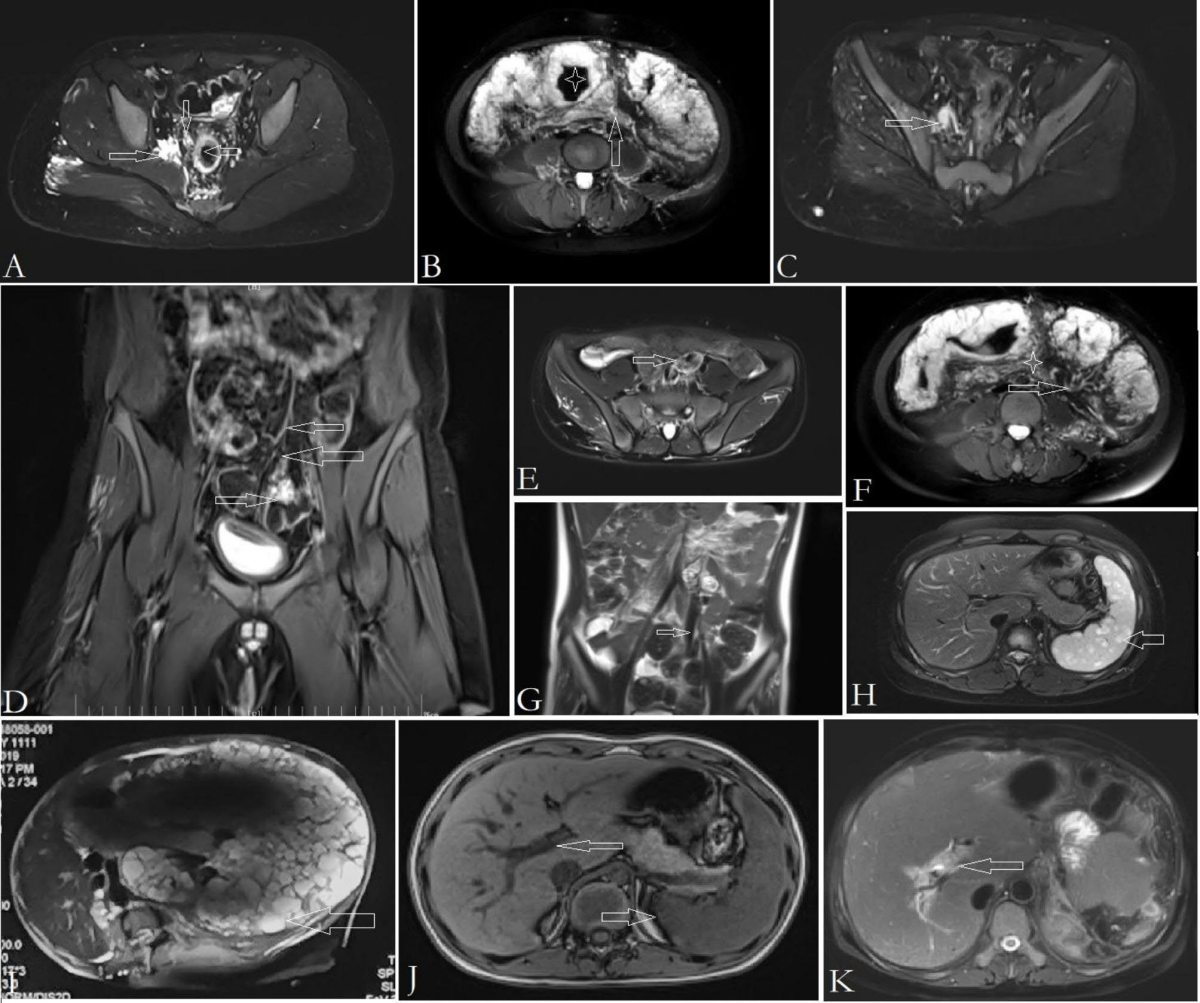
MRI findings in KTS with anorectal and anorectosigmoid VM. Panel **A**: Dilated, incompetent middle rectal vein (rightward arrow), perirectal VM (downward arrow), and thickening of rectal wall by VM involving (leftward arrow). Panel **B**: Sigmoid lumen became narrowed by thickening of the wall (asterisk). The mesentery of sigmoid colon become thickened and edematous (arrow). Panel **C**: Dilated, incompetent IIV (rightward arrow) is demonstrated. Typical fluid signal in the lumen is indicative of stagnation and/or reflux of blood. Panel **D**: Part of VM in rectosigmoid colon (rightward arrow) drains into the superior rectal vein (leftward arrows). Panel **E and G**: Ectatic and incompetent inferior mesenteric vein is identified (arrow). Heterogeneous signal in the lumen is indicative of blood stagnation, turbulent flow and reflux. Panel **F**: Dilation and tortuosity of the sigmoid vein in the mesentery (arrow). It drains into inferior mesenteric vein. Thickening and edema of the sigmoid mesentery also can be identified (asterisk). Panel **H and I**: Multiple cystic lymphatic malformations within the spleen (arrow). Splenectomy is indicated for massive splenomegaly (Panel **I**). Panel **J**: Main branches of portal vein become dilated (leftward arrow), and spleen enlarged (rightward arrow), which is suggestive of portal hypertension, although obstruction is not evident. Panel **K**: Main branches of portal vein become dilated and obstructed (arrow). Circumferential edema is notable. The spleen was resected because of portal hypertension and massive splenomegaly by lymphatic malformations

In KTS patients with cavernous transformation of the portal vein, however, this shunt is inadequate [3]. The initial obstruction or thrombosis of portal vein occurs in small portal branches, not in a segment of trunk or main branches [3]. This is a form of non-cirrhotic intrahepatic portal hypertension. In this form of portal hypertension, thrombi are diffused and even non-evident (Fig. 4). Extensive cavernous transformation of the portal vein in liver and massive obstruction in porta are the later features (Fig. 4). Portal decompression cannot be achieved by mesenterico-Rex shunt in these patients [3].

In KTS patients the small branches are obstructed, subsequently the intrahepatic portal branches are dilated in early stage of portal hypertension (Fig. 2). As the VM develops with age, main branches of portal vein become obstructed, portal hypertension deteriorates, and subsequent extensive cavernous transformation of portal vein in liver and collateral channels develop in porta [35]. Thus, the portal hypertension in KTS has a distinct pathophysiology from common extrahepatic portal vein obstruction in children. Mesenterico-Rex shunt is not suitable for portal hypertension in children with KTS involving gastrointestinal tract (Fig. 3).

#### “Hemorrhoids” is not true hemorrhoids in KTS with gastrointestinal involvement

In appearance, anorectal VM looks like hemorrhoids, but is not true hemorrhoids. It is usually a sign of IIV malformation and reflux [2, 6]. “Hemorrhoids” can be also just the tip of the iceberg of gastrointestinal involvement and/or portal hypertension in KTS [6]. Consequently, indiscriminate sclerotherapy should be avoided to manage “hemorrhoids” (Fig. 3).

#### Splenectomy can be indicated for massive splenomegaly in KTS

In KTS patients, splenic lesions is multifocal or diffuse lymphatic malformations [1, 3], not so called “hemangioma”. Splenomegaly also can be presented in KTS, but there is often no or little clinical significance. Occasionally, splenomegaly causes significant mass effect and/or hypersplenism (Fig. 4). Splenectomy can be indicated for massive splenomegaly. Splenic artery embolization is ineffective for lymphatic malformations [36].

#### ISSVA classification of vascular anomalies is recommended for gastroenterologist to report gastrointestinal involvement in KTS

Until know, there are confusing terms to describe the lesions involving the gastrointestinal tract in KTS, such as hemangioma [11, 23, 48], cavernous hemangioma [20], lymphangioma [17, 23], and varicosities [20, 26] etc. Terms from the International Society for the Study of Vascular Anomalies (ISSVA) are recommended to use in reports [49]. In this classification, the term “hemangioma” refers to some true vascular tumors, including infantile hemangioma, congenital hemangioma, spindle-cell hemangioma, and epithelioid hemangioma, et al. [5, 49, 50]. However, vascular component involved in the gastrointestinal tract is VM, or occasionally lymphatic malformation, not tumor. Endoscopic and radiologic findings are consistent with VM [5] involving gastrointestinal tract in KTS reports. Histopathologically, VM consists of dilated, thin-walled, sponge-like abnormal channels [5] (Fig. 2), but tumor cells are absent. Since 1982 vascular tumors have been differentiated from vascular malformations based on endothelial characteristics [51]. They have different clinical history, imaging and histopathological characteristics, and biological behavior [51]. The words “cavernous hemangioma,” “lymphangioma” are erroneous when used for VM [5], and thus the ISSVA has discarded these terms since its 1996 workshop [5], in order to give us a common language.

#### Basic MRI sequences is recommended to evaluate gastrointestinal involvement in KTS

In many case reports of KTS, the gastroenterologists preferred computed tomography (CT) to evaluate gastrointestinal involvement, but the provided information was very limited. It is difficult to identify VM and dilated veins on CT scan, even with contrast enhancement. Because of relatively quiescent and stagnant flow within these malformative veins, the contrast entering lesions is very little and poorly diffuses, and these lesions cannot be well enhanced and demarcated on CT.

KTS is a complex low-flow vascular malformation with absence of arterial component. So basic magnetic resonance imaging (MRI) sequences is preferred to evaluate most of the findings in KTS [1, 3]. In KTS patients with gastrointestinal involvement, the findings on MRI sequences include thickening of the anorecto(sigmoid) colon by circumferential and intra-walled VM, edema and thickening of the affected mesentery, malformations and incompetency of IIV and IMV systems, dilation of mesenteric veins, and possible lymphatic malformation of spleen (Fig. 4). Dilation and/or occlusion of portal vein and branches also can be identified on MRI sequences (Fig. 4).

VM of pelvis and colon, and lymphatic malformation of spleen feature a typical fluid signal on T2-weighted MRI sequences [3] (Fig. 4). Incompetency of the involved veins features a higher fluid signal than that in normal veins on T2-weighted MRI sequences [2, 3] (Fig. 4). Dilation of these veins is an indication of the presence of reflux [2]. In our study, basic MRI sequences is highly sensitive to detect the IIV reflux in KTS with pelvis involvement; the sensitivity was as high as 97.56%^2^. Dilation and tortuousity of mesenteric veins in the affected area also can be demonstrated on MRI (Fig. 4). Additionally, phlebolith is charactered by local low signal within VM (Fig. 4).

These findings on MRI sequences represent most of the changes of gastrointestinal involvement in KTS patients, including structural details and hemodynamic characteristics of veins. However, it is difficult to provide these information by CT scan. So, we recommend basic MRI sequences to evaluate most of the gastrointestinal findings in KTS patients. CT can be used for diagnostic confirmation and to detect the extension of portal venous obstruction [43].

## Conclusion

KTS is typically a combined slow-flow vascular malformations (capillary, lymphatic, and venous) in an overgrown lower limb. In current literature and our study, gastrointestinal involvement in KTS mainly refers to anorectal and anorectosigmoid VM. They have different pathophysiologies. The former has the malformation and abnormal hemodynamics of IIV system. The latter involves the malformation and abnormal hemodynamics of two vein systems, IIV and IMV, can cause portal hypertension.

In KTS patients with pelvis involvement, the IIV-gluteal-marginal vein system is a valveless malformed reflux channel, which can cause bleeding from pelvic organs (anorectum and genitourinary tract). Anorectal VM and its underlying IIV reflux can be managed by ethanol-based sclerotherapy, hybrid multidisciplinary approaches, and/or trans-anal endorectal pull-through.

When sigmoid colon involved in KTS patients, evaluation and management become more complicated. Comprehensive assessment of pelvis and abdomen is recommended, especially the hemodynamic characteristics of rectal VM and IMV. Basic MRI sequences is preferred to evaluate the gastrointestinal findings in KTS patients. CT can be used to confirm and detect the extension of portal venous obstruction. Recognizing the distinct pathophysiology is required to prevent pitfalls in the management. Staged surgeries and intervention can be planned in KTS patients with gastrointestinal involvement.

This review discusses the pathophysiology in anorectal VM and highlights the distinctiveness of anorectosigmoid involvement and portal hypertension in KTS. We recommend MRI as an important evaluation method and point out pitfalls in management, and propose treatment strategies for this rare disease. Nevertheless, the pathophysiology regarding portal hypertension in KTS we discussed should be considered in future guidelines for vascular diseases of the liver. Despite our efforts to integrate the current knowledge regarding the gastrointestinal involvement in KTS, our analysis is based on low level of evidence from a small number of patients, and the conclusions might be controversial.

## Data Availability

No datasets were generated or analysed during the current study.

## Abbreviations

KTS: Klippel-Trénaunay syndrome
VM: venous malformation
IMV: inferior mesenteric vein
IIV: internal iliac vein
LIC: localized intravascular coagulopathy
DIC: disseminated intravascular coagulation
ISSVA: International Society for the Study of Vascular Anomalies
CT: computed tomography
MRI: magnetic resonance imaging
MSV: median sacral vein
SRV: superior rectal vein
LM: lymphatic malformation
